# Study on the Expansion Dynamics of MDCK Epithelium by Interstitial Flow Using a Traction Force-Measurable Microfluidic Chip

**DOI:** 10.3390/ma14040935

**Published:** 2021-02-16

**Authors:** Mirim Kim, Hwanseok Jang, Yongdoo Park

**Affiliations:** Department of Biomedical Sciences, College of Medicine, Korea University, Seoul 02841, Korea; mrkmoya@korea.ac.kr (M.K.); kevin14@korea.ac.kr (H.J.)

**Keywords:** fluid flow, collective cell migration, MDCK, traction force microscopy, monolayer stress microscopy, microfluidics

## Abstract

The movement of collective cells is affected through changes in physical interactions of cells in response to external mechanical stimuli, including fluid flow. Most tissues are affected by fluid flow at the interstitial level, but few studies have investigated the physical effects in collective cells affected by a low flow rate. In this study, collective cell migration of Madin–Darby canine kidney (MDCK) epithelial cells was investigated under static or interstitial flow (0, 0.1, and 1 μL/min) using a traction microfluidic device. The optimization of calculation of cellular traction forces was first achieved by changing interrogation window size from the fluorescent bead images. Migration analysis of cell collectives patterned with a 700 μm circular shape reveals that cells under the slow flow (0.1 and 1 μL/min) showed the inhibitory migration by decreasing cell island size and cellular speed compared to that of static condition. Analysis of cellular forces shows that level of traction forces was lower in the slow flow condition (~20 Pa) compared to that of static condition (~50 Pa). Interestingly, the standard deviation of traction force of cells was dramatically decreased as the flow rate increased from 0 to 1 μL/min, which indicates that flow affects the distribution of cellular traction forces among cell collectives. Cellular tension was increased by 50% in the cells under the fluid flow rate of 1 μL/min. Treatment of calcium blocker increased the migratory speed of cells under the flow condition, whereas there is little change of cellular forces. In conclusion, it has been shown that the interstitial flow inhibited the collective movement of epithelial cells by decreasing and re-distributing cellular forces. These findings provide insights into the study of the effect of interstitial flow on cellular behavior, such as development, regeneration, and morphogenesis.

## 1. Introduction

The collective migration of cells is a form of harmonized behavior required for critical life activities such as development [1,2] and regeneration [3] as well as for cancer metastasis [4]. Tissues and organs consist of physically connected cells that move by exerting mechanical forces in response to their extracellular environment, which includes mechanical stimuli [3,5,6]. One type of mechanical stimulus is fluid flow that modulates the functions of cells and organs in the body. The effects of fluid flow on the body, from the interstitial to aortic levels, are highly dependent on the fluid flow rate. For higher flow rates, such as in the aorta and vena cava, ranging around 20 dyn/cm^2^ [7], studies are focused on the endothelial cells showing changes in morphological and physiological aspects, such as alignment of cytoskeletal structure [8,9] and the induction of genes related to shear stress [10]. For lower flow rates, such as capillary flow [11], studies are focused on the integrity of the endothelial cell monolayer related to controlling permeability [12]. Low flow rates also exist in the epithelium when building tissue lumen or ducts. For example, flow in the renal tubule leads to junction remodeling [13,14] and protein redistribution [15]. Corneal epithelial cells exposed to low flow rates exhibited increased migration in a wound healing model [16].

Interstitial flow, showing an extremely slow flow rate, is another way body fluid is encompassed within cells and the extracellular matrix of tissues. Although the basic role of interstitial flow is nutrient supply and circulation, many studies have extensively shown that it has different effects depending on cell type. As a morphogenetic regulator, it helps in lymph vessel development by mediating lymphatic endothelial cell migration [17]. With regard to pathology, interstitial flow affects tumor cell proliferation, invasion [18], and myofibroblast differentiation [19]. Low shear stress exerted by interstitial flow induces differentiation of stem cells [20] and enhances maturation of tubular epithelial cells [21]. In addition, it helps intestinal epithelial cells on a chip to mimic in vivo structures [22]. As mentioned, although various changes in cells are known to be caused by interstitial flow, studies on the physical response of cells are lacking.

The integrity of the epithelial monolayer is important in understanding both the physiology of organ function and the biofunction of individual cells. A physical force map indicates the dynamics of cellular tension and traction in the epithelial cell monolayer. For the quantification of multicellular force, many researchers have used traction force microscopy (TFM) with Madin–Darby canine kidney (MDCK) 2D sheet models [23]. One study that analyzed the physical relevance of the movement of multicellular units showed the balance in the internal force of moving epithelial cells. These collective movements were described as being in a tug of war, in that the connected long tensile force of locally generated forces throughout the island regulates the movement of the whole island [24]. In addition, it was shown that the collective cell migration patterns were the result of feedback through cell–cell interactions through the collective migration study according to various geometric cell cluster structure [25]. In a wound healing model, it was described that tension was transmitted through heterogeneous cytoskeletal structure [26]. The understanding of epithelial collectiveness has been further improved, but the variation of forces resulting from multicellular units by external stimulation remains unknown.

In this study, we implemented an experimental platform that integrates TFM technology with microfluidic chips to determine how interstitial flow affects the cohesiveness of the epithelial monolayer. Using this system, we were able to measure the collective movements of the circular patterned MDCK monolayer and the cellular forces in static and interstitial flow conditions. The measured raw data were quantitatively analyzed through bio-image optimization. The expansion ratio of cell island and the cell speed were represented in graphs over time, and pathway with distance was shown as cellular trajectories. Traction exerted in the underlying matrix and the tension generated between cell interfaces were mapped to analyze spatial distribution, and the mean force of each group in time was plotted. As a result, it can be seen that interstitial flow inhibited the mobility of MDCK cells and altered the physical dynamics of cell islands, and the inhibition of calcium influx has been confirmed to alter cell migration and force distribution.

## 2. Materials and Methods

### 2.1. Cell Culture

Madin–Darby canine kidney (MDCK; strain II) cells were cultured in T75 flasks (SPL, Phocheon, Korea) containing Dulbecco’s Modified Eagle’s Medium (DMEM, Gibco, Grand Island, NY, USA) with 10% fetal bovine serum and 1% penicillin and streptomycin. The cells were maintained at 37 °C and 5% CO_2_.

### 2.2. Sample Preparation

#### 2.2.1. Preparation of Polyacrylamide Gel-Filled Bottom Glass

The procedure for preparing polyacrylamide (PAA) gels is described in our previous paper [27]. In brief, pre-cured PAA gel solution (Bio-Rad, Hercules, CA, USA) containing fluorescent microbeads (Invitrogen, Carlsbad, OR, USA) was placed on the silane-coated surface of a rectangular etched customized glass (Amed, Seoul, Korea) with a depth of 100 μm and covered with 18 mm ϕ cover glass (Marienfeld-Superior, Lauda-Konigshofen, Germany). The bottom glass, filled with PAA gel solution, was placed upside down in a dark place for 45 min at room temperature. After gelation of the PAA gel, the cover glass was removed and the gel’s surface was activated by sulfosuccinimidyl 6-(4′-azido-2′-nitrophenylamino)hexanoate (Sulfo-SANPAH, Thermo Scientific, Waltham, MA, USA) treatment with UV light (365 nm wavelength; UVP LLC, Upland, CA, USA) and coated with 100 μg/mL of collagen solution (Corning, Corning, NY, USA).

#### 2.2.2. Fabrication of Polydimethylsiloxane (PDMS) Stencil and Microfluidic Channel

Stencils for cell patterning and top chips for the microfluid channel were fabricated by soft lithography using polydimethylsiloxane (PDMS; Silgard 184, Dow Corning, Midland, MI, USA). SU-8 master mold for stencil with posts (diameter = 700 μm) and microfluidic channels with tree structures were customized by Amed, Seoul, Korea. PDMS prepolymer solution (10:1 ratio) was poured into the master mold and cured at 85 °C for 2 h after removing air bubbles in a desiccator. PDMS stencils with holes were trimmed with a 14 mm-diameter punch. The microfluidic channel parts, with dimensions 12 mm × 6 mm × 160 μm (length × width × height) were cut out to 24 mm × 24 mm and both ends of the tree structure were punched with a 1 mm-diameter biopsy punch (Integra Miltex, York, PA, USA) for an inlet and outlet of fluid flow (Figure S1). All PDMS parts were autoclaved before use.

#### 2.2.3. Patterning Cellular Islands

PDMS stencils were coated with Pluronic F-127 solution (Sigma-Aldrich, St. Louis, MO, USA) in PBS (Biowest, Nuaillé, France) at 37 °C for 1 h and washed with phosphate buffered saline (PBS). After washing the collagen-coated PAA gel with PBS, stencils were placed on top so that 12 holes were placed in the center of the gel. The stencil holes were filled with PBS to remove air bubbles and covered with a 200 μL droplet of MDCK cell suspension DMEM media (density = 1 × 10^6^/mL). After 1 h of incubation, the remaining cell debris on the islands was washed out with fresh media. Then, stencils were removed and 4 ml DMEM was added to each sample.

### 2.3. Assembly of Microfluidic TFM System

The microchannel-engraved side of the prepared PDMS part was treated with an oxygen plasma system (Femto Science, Hwaseong, Korea) for hydrophilic surface modification. After covering the bottom glass part that had the patterned cell island array with the plasma-treated PDMS chip, the integrated part was fixed with a customized holder (Han-Gug Mechatronics, Seoul, Korea). A yellow pipet tip filled with 200 μL of media was fixed to the inlet and an empty yellow pipet tip was inserted into the outlet with gentle suction to fill the channel with media and remove air bubbles. A syringe cylinder (KOVAX, Seoul, Korea) with the piston removed served as a reservoir and was inverted and connected to tubing consisting of a 3-way stopcock (Hyupsung, Seoul, Korea), a 30 cm volume line (Hyupsung, Seoul, Korea), and a trimmed 18 G needle (KOVAX, Seoul, Korea), and was then filled with fresh media and connected to the inlet of the chip. A syringe was connected to the outlet of the chip through tubing consisting of a 3-way stopcock, a 75 cm volume line (Hyupsung, Seoul, Korea), and a trimmed 18 G needle, then filled with fresh media and installed on a syringe pump (Chemyx Inc., Stafford, TX, USA). The fluid flow was generated at negative pressure using the syringe pump unidirectionally in the laminar condition. The experiment was conducted for a total of 600 min under 3 conditions of varying flow rate: 0 (static), 0.1, and 1 μL/min. For experiments where calcium ion influx was inhibited, ruthenium red (Sigma Aldrich, St. Louis, MO, USA), a calcium channel blocker, was added to the inlet reservoir at a final concentration of 30 μM.

### 2.4. Time-Lapse Microscopy

All experiments were operated on a JuLI-stage live cell imaging system (NanoEnTek In., Seoul, Korea) at 37 °C with 5% CO_2_ incubator. Cells were imaged using bright-field microscopy, and green fluorescent protein (GFP) filters were used to image fluorescent beads. The cell and bead images were captured every 10 min for 10 h. After imaging, the cells were detached from the PAA gel by trypsin treatment to obtain reference bead images for analysis.

### 2.5. Quantification Analysis

Phase contrast cell and fluorescent bead images obtained by time-lapse microscopy were computed to quantify cellular motility and force using the customized MATLAB code used in previous studies [27,28]. Briefly, at each time point, the cell image and its next cell image or bead image and the reference bead image were calculated using a particle image velocimetry (PIV) algorithm based on cross-correlation (calculation of correlation between serial cell images). Displacement vectors from cell images were converted to cell velocity maps and trajectories. Traction force maps were obtained from displacement vectors of fluorescent bead images through unconstrained Fourier transform traction microscopy (FTTM). The traction data were converted to intercellular stress within a cell island using monolayer stress microscopy (MSM).

## 3. Results

### 3.1. Integrated Traction Force Microscopy with Microfluidic System

**Figure 1:**
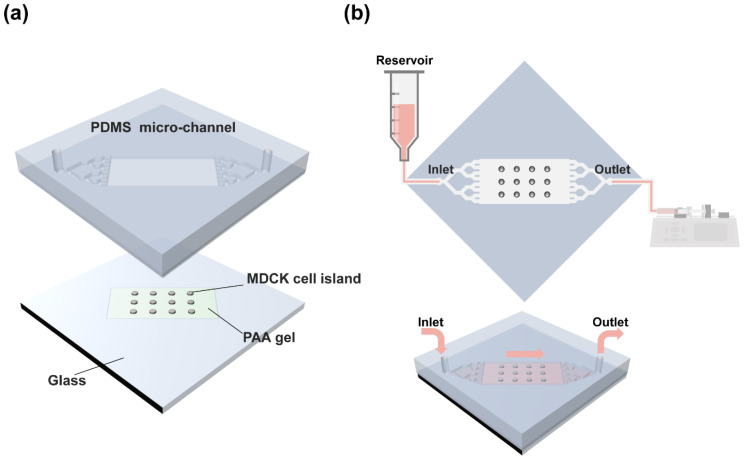
Schematics of assembly of the traction force-measurable microfluidic device and generation of fluid flow using a syringe pump. (**a**) Assembly of top polydimethylsiloxane (PDMS) microchannel part and bottom glass part. On the glass part, circular Madin–Darby canine kidney (MDCK) cell islands are patterned on the polyacrylamide (PAA) gel with embedded fluorescent beads for traction force measurement. (**b**) The syringe cylinder, serving as a reservoir, is connected to the inlet of the traction force microfluidic device, and a syringe pump controlling the flow rate by fluid withdrawal speed is connected to the outlet of the device (upper panel: top view, lower panel: bird’s-eye view).

To analyze the effect of fluid flow on collective cellular movement from a physical perspective, we used a combination of microfluidic chip and traction force microscopy (Figure 1). Both the inlet and outlet sides of the PDMS microchannel (top part) were designed in a tree structure to allow for a flow rate throughout the chip (Figure 1a). The two-dimensional cellular migration model was set as a circular pattern of MDCK cells, which provides the benefits of being able to observe unbiased movement and the collectiveness of the entire cell island. The circular pattern size was determined to be 700 μm in diameter and capable of maintaining collective movement in a cellular monolayer island rather than characterized by dissociated single-cell motion [29]. The standardized cell islands were patterned on the PAA gel in customized glass and the microchannel-engraved PDMS chip was put on top of the glass to complete the closed channel. Stable laminar fluid flow was generated in one direction from the inlet connected to a syringe cylinder as a reservoir to the outlet by withdrawing the fluid using a syringe pump with negative pressure (Figure 1b). Multi-physics simulation was used to confirm maintenance of laminar flow in fluidic chip (Table S1, Figure S1). As a result, this integrated system enabled measurement of the physical forces generated by cells exposed to various fluid flow environments.

### 3.2. Evaluation of Particle Image Velocimetry (PIV) Analysis Results According to Size of Interrogation Window (IW) and Application of Fluid Flow

**Figure 2:**
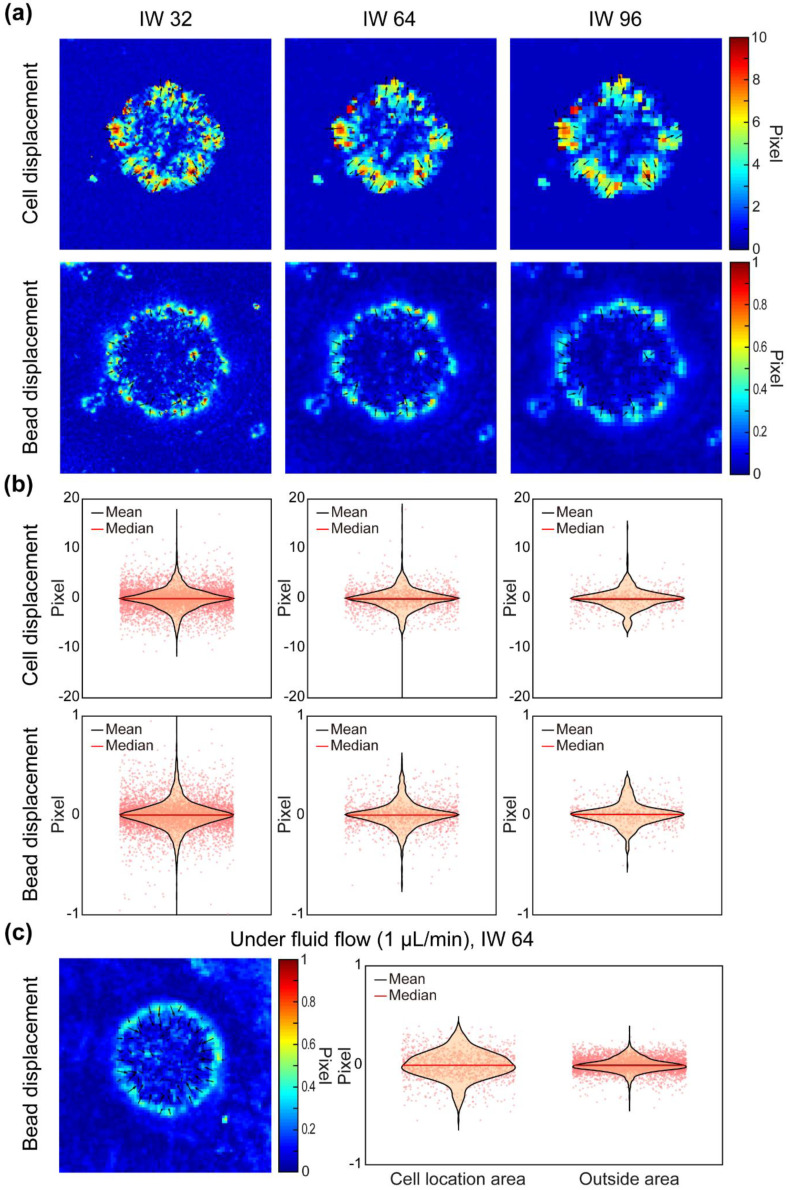
Results of particle image velocimetry (PIV) analysis by interrogation window (IW) size and application of fluid flow. (**a**) Spatial resolution of PIV analysis results from cell (upper panels) and bead (lower panels) images according to 3 IW sizes is described using color-coded maps (IW 32, IW 64, IW 96: 32 × 32, 64 × 64, 96 × 96 pixels/window, respectively). (**b**) Data distribution of cell (upper panels) and bead (lower panels) displacement from area of cell location according to IW size is described by violin plots with jitter (black line: mean, red line: median). (**c**) Error of bead displacement due to application of fluid flow is evaluated by color-coded map of entire region and violin plots with jitter in the location and outside regions.

The quantitative analysis of cellular motility and forces requires cross-correlation-based PIV analysis from cell and fluorescent bead images. In this procedure, the displacement of particles (i.e., cells or beads) is calculated by assessing the cross-correlation of a specified interrogation window (IW) size. Therefore, optimization of the IW size is critical to obtain accurate and efficient analysis results. To find an appropriate IW size, the particle displacement results from the cell and bead images according to three IW sizes (32 × 32, 64 × 64, and 96 × 96 pixels) were evaluated for spatial resolution through color-coded displacement maps, and for data distribution, through violin plots with jitters (Figure 2). As shown in the color-coded displacement map, the larger the IW size, the lower the spatial resolution and the more data are lost by over-smoothing (Figure 2a). On the other hand, the smaller the IW size, the greater the spatial resolution, but the greater the consumption of time and memory space for analysis. Even if the IW size is excessively small, noise can also be generated by deviating from the correlation analysis.

In Figure 2b, the violin plots with jitter dots indicate the distribution of IWs presenting displacement values along the x-axis direction (left, negative; right, positive) within the cell island area. In measuring cell displacement, IW 32 showed excessively concentrated values near 0 in the center, IW 64 detected the widest range of displacement, and IW 96 showed a remarkable loss of data points. In measuring the bead displacement, IW 32 detected the widest range of displacement but still showed excessively concentrated values near 0 in the center, IW 64 showed less range and concentration of data points than IW 32, and IW 96 showed the lowest data number and smallest range. As a result, IW 32 is the best IW size for analyzing bead displacement and IW 64 for analyzing cell displacement; however, we used IW 64 for both PIV analyses of cell and bead displacement to match data points between motility and force data, and to save time and memory consumption.

Additionally, we confirmed whether fluid flow affects bead displacement using IW 64 (Figure 2c). There was no overall effect of bead displacement under the fluid flow in terms of which direction was left to right in the color-coded map. However, cells could be affected by the flow. Thus, we plotted the data distribution of the cell island location area and the outside area with violin plots and jitter dots, respectively. The bead displacement within the cell island location area showed a non-ideal balance distribution. Still, one outside area from the cell island location showed balanced and concentrated distribution centered around 0. This result indicates that the flow rate used in this study did not affect the bead displacement or cellular force measurement.

### 3.3. Fluid at the Level of Interstitial Flow Rate Attenuates Expansion of MDCK Cell Islands

**Figure 3:**
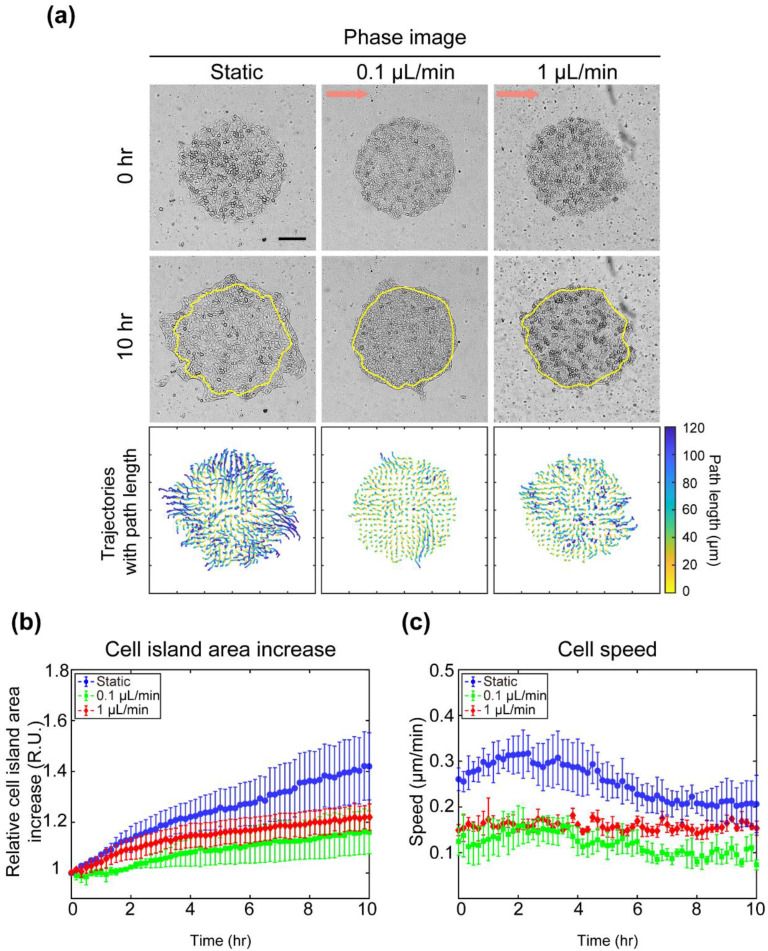
Change in MDCK cell island expansion and cell migration according to flow rate. (**a**) Phase images of MDCK cell islands captured at 0 and 10 h under each flow rate condition (static, 0.1 μL/min, and 1 μL/min). Flow direction is left to right (arrow). Yellow line represents initial cell island boundary. Scale bar = 200 μm. Cell trajectories of cell islands for 10 h under each flow rate condition colored according to path length. (**b**) Average area increase of cell islands under each flow rate condition plotted over 10 h (blue circle: static; green square: 0.1 μL/min; red triangle: 1 μL/min). Error bars indicate standard deviation (n = 3). (**c**) Average cell speed of cell islands under each flow rate condition plotted over 10 h (blue circle: static; green square: 0.1 μL/min; red triangle: 1 μL/min). Error bars indicate standard deviation (n = 3).

To explore the effect of interstitial flow on MDCK collective cell migration, MDCK cell movement within a cell island was observed for 10 h under three flow rate conditions: 0 (static), 0.1, and 1 μL/min (Figure 3). Under the static condition, circular patterned cells radially expanded into free space, showing irregular finger-like formations at the edge (Figure 3a). However, when interstitial flow was applied, the expansion of cell islands to free space was reduced. To clarify the difference in the path of cellular motion among cell islands under each flow condition, the trajectory was calculated using cell displacement data (Figure 3a). Lines show the path of each cell, and the color of the path indicates its length. Most cells under the static condition migrated over 100 μm (dark blue) for 10 h. On the other hand, cells under 0.1 and 1 μL/min conditions migrated below 60 and 80 μm, respectively. In addition, the change in cell island area and cell speed under each condition was statistically compared over time (Figure 3b,c). The cell island area showed the steepest increase under the static condition, up to around 1.5-fold, but a moderate increase under the condition of interstitial flow, up to around 1.2-fold (Figure 3b). Under the static condition, the cell speed of the island fluctuated between 0.2 and 0.3 μm/min until 6 h after the start of expansion (Figure 3c). After 6 h, it plateaued at around 0.2 μm/min. The cell speed under the flow condition of 0.1 μL/min showed the same fluctuation but half the magnitude of the one under the static condition over time. Under the flow condition of 1 μL/min, the cell speed was maintained at 0.15 μm/min for 10 h. These results indicate that the presence of interstitial flow attenuates MDCK cell island expansion by reducing cell speed.

### 3.4. Interstitial Flow Changes the Physical Dynamics of Cell Islands

**Figure 4:**
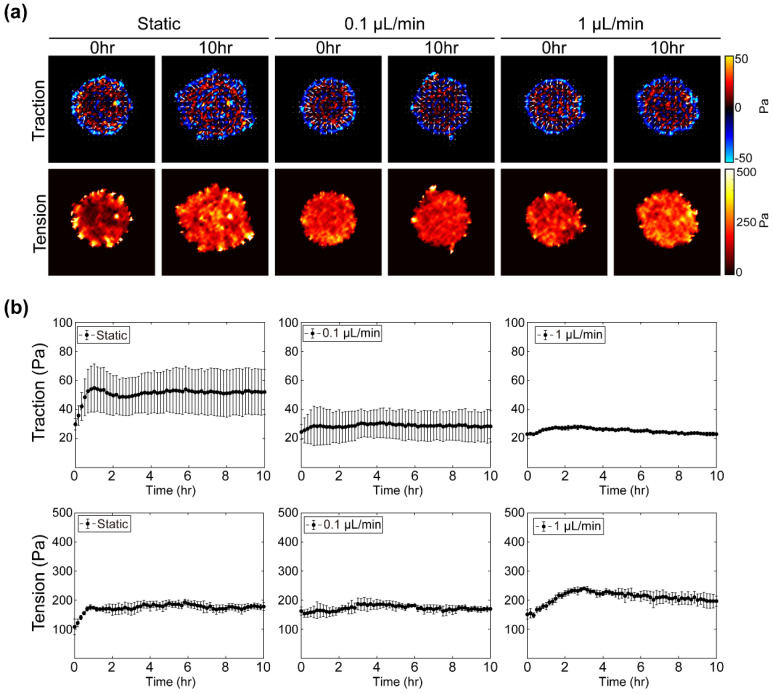
Cell–substrate and cell–cell physical responses of cell islands under each flow rate condition. (**a**) Traction maps of cell islands at 0 and 10 h under each flow rate condition colored according to force strength in radial coordination (red: outward directional forces from the center; blue: force toward the center). White arrows indicate direction of cellular traction forces. Tension maps of cell islands at 0 and 10 h under each flow rate condition colored according to strength of average normal stress (dark to bright). (**b**) Average traction of cell islands under each flow rate condition plotted over 10 h. Error bars indicate standard deviation (n = 3 or 4). Average tension of cell islands under each flow rate condition plotted over 10 h. Error bars indicate standard deviation (n = 3 or 4).

Cell movements are governed by cell–cell and cell–substrate interactions. Therefore, in order to understand the difference in cell expansion seen in each flow condition from a physical point of view, we analyzed the cellular traction force and tensional stress of the cell islands under each condition over space and time (Figure 4). Color-coded maps identify the spatial distribution of cellular traction and tension at initial and final time points (Figure 4a). In the static condition, both traction and tension were highly concentrated on the edge of the cell island at the beginning of expansion. Ten hours later, an irregular finger that formed at the edge still exerted high-strength traction and tension and a high magnitude of forces developed inside the cell island. Under 0.1 μL/min flow condition, the initial traction force was distributed isotropically toward the center along the edge of the cell island, and the initial tension was homogeneously generated over the cell island. Even after 10 h, strong forces were partially generated on the periphery, but the magnitude and homogeneity of the force were sustained throughout the cell island. In 1 μL/min flow condition, the strength and distribution of the initial forces were comparable with those of the 0.1 μL/min condition, but after 10 h, the tension at the inside and periphery of the island were comparatively increased. Statistical variations in cellular traction and tension over time were plotted with averages and deviations using 3 or 4 samples for each condition (Figure 4b). In the static state, average traction and its deviation were maintained at around 50 Pa after the initial fluctuation for 4 h. Under the condition of 0.1 μL/min, it was maintained at around 30 Pa without a large fluctuation over time. For the 1 μL/min flow condition, the traction force decreased to about 20 Pa after the initial gentle fluctuation for 2 h. Average tension in the static state initially increased for an hour and then maintained at about 200 Pa. For 0.1 μL/min, an initial average tension of 200 Pa was maintained to the end. For 1 μL/min, average tension was initially 150 Pa, similar to that for 0.1 μL/min, but increased to 230 Pa (~ 50% increase) for 3 h, then gradually decreased and dropped to 200 Pa in the last 10 h. Overall, as cells were exposed to the interstitial flow, spatial heterogeneity of traction and tension of the cell monolayer was decreased, and the intra-group deviation of average traction decreased, but average tension was somewhat increased (Figure S2).

### 3.5. Inhibition of Calcium Influx of MDCK Cells Exposed to Fluid Flow Alters Motility and Force Distribution

**Figure 5:**
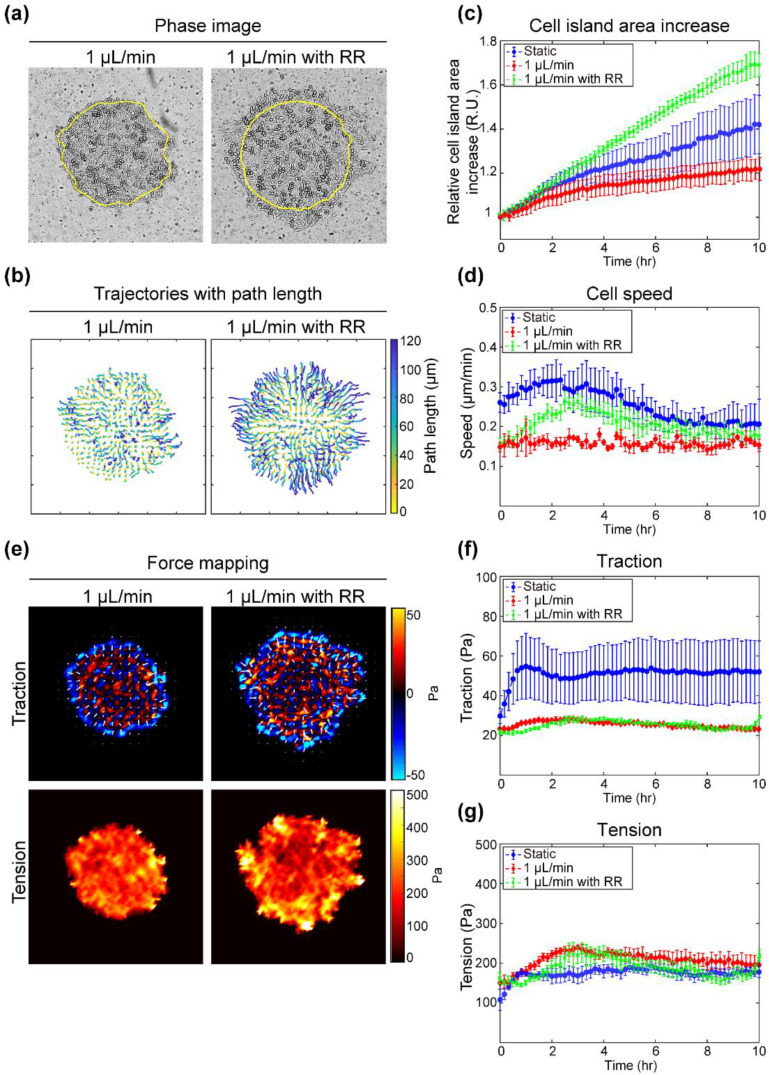
Comparison of response of cell islands to inhibition of the calcium ion influx induced by fluid flow with that of uninhibited cell islands under static or fluid flow conditions. (**a**) Phase images of MDCK cell islands captured after 10 h under each condition (left: 1 μL/min; right: 1 μL/min with 30 μM of ruthenium red (RR)). Yellow line represents initial cell island boundary. Scale bar = 200 μm. (**b**) Cell trajectories of cell islands for 10 h under each condition colored according to path length. (**c**) Average area increase of cell islands under each condition plotted over 10 h (blue circle: static: green cross: 1 μL/min with RR; red diamond: 1 μL/min). Error bars indicate standard deviation (n = 3 or 4). (**d**) Average cell speed of cell islands under each condition plotted over 10 h (blue circle: static; green cross: 1 μL/min with RR; red diamond: 1 μL/min). Error bars indicate standard deviation (n = 3 or 4). (**e**) Traction (upper panels) and tension maps (lower panels) of cell islands after 10 h under each condition colored according to strength. White arrows indicate direction of cellular traction forces. (**f**) Average traction (upper panel) and (**g**) tension (lower panel) of cell islands under each condition (blue circle: static; green cross: 1 μL/min with RR; red diamond: 1 μL/min) plotted over 10 h. Error bars indicate standard deviation (n = 3 or 4).

A recent study reported that the influx of calcium ions has a significant influence on epithelial cell physiology [30,31]. However, since there is a lack of research regarding whether interstitial flow affects calcium influx, we investigated whether the results of cellular motility and force change by the slow flow rate are due to the influx of calcium ions. To inhibit the ability of calcium influx under flow conditions, ruthenium red (RR) was added to media while maintaining flow at 1 μL/min (Figure 5). Under 1 μL/min flow, the addition of RR resulted in increased cell island expansion and appearance of irregular boundary shape compared with no RR addition (Figure 5a). The trajectory of the RR-treated island also indicated that most of the cells migrated more than 100 μm, as shown in dark blue (Figure 5b). In addition, the increase in cell island areas of the static, 1 μL/min, and RR-treated 1 μL/min groups was statistically compared over time (Figure 5c). The cell island expansion under 1 μL/min with RR increased by more than 1.6 times compared to the initial area, which was 20% and 40% higher than for the static and 1 μL/min groups, respectively. Average speed for 1 μL/min with RR was the same as the 1 μL/min group at the beginning and end and lower than the static group (Figure 5d). The profile of cellular speed over time of the RR group showed a similar tendency to that of the static group. In short, blocking calcium ion flow increased cell expansion and the cell migration speed even in the presence of flow. Moreover, in the cellular force maps, the 1 μL/min condition with RR added showed increased traction and tension at the island periphery and these were decreased inside the island (Figure 5e). Even the distribution of traction and tension within the cell island under flow was returned to its heterogeneous state by RR treatment. As confirmed in Figure 4, the fluid flow presence suppressed the magnitude of traction force and reduced the deviation of average value between samples (Figure 5f). However, under flow conditions, the average magnitude and variation of traction were not affected by the suppression of calcium influx through RR treatment. Interestingly, whereas the average tension in the static and 1 μL/min groups stabilized within 2–3 h at different magnitudes, the 1 μL/min with RR group showed average tension fluctuations around the ranges of tension magnitude in the groups over time (Figure 5g). As a result, inhibiting calcium influx in the presence of interstitial flow caused the overall traction force to still be lower under flow, but the intercellular tension fluctuated around low values. In the meantime, traction and tension at the edge remained high, allowing cells at the edge to increase migration.

## 4. Discussion

Collective cell migration is a harmonized cellular response to external physicochemical stimuli and is required for development and morphogenesis as well as physiological homeostasis [32]. Since observation of the dynamics of these cell clusters is limited using conventional biological methods, biophysical methods, such as TFM, have allowed the mechanisms of collective cell behavior to be studied and more clearly understood [33]. In this study, in order to quantify the dynamics of the movement and physical force of collective cells under a fluid flow environment, a traction force-measurable microfluidic chip was developed by improving upon the system developed in our previous study [28]. The microchannel in the inlet and outlet was designed in the shape of a tree branch such that a constant flow rate was evenly distributed over the cell islands arranged in the channel. This made it possible to simultaneously test several cell island samples under the same flow rate conditions. In addition, using the cell and fluorescent bead images obtained through this system, PIV analysis with an appropriate IW window enabled analysis by correlating the spatiotemporal distribution of cell motion and force. Additionally, it was also confirmed that the wall shear stress of the maximum flow velocity (1 μL/min) applied in this experiment did not cause fluorescence bead displacement when measuring the traction force. Therefore, we were able to secure a system capable of measuring epithelium dynamics under interstitial flow conditions.

MDCK cells are a representative mammalian epithelial cell line used to study a variety of processes in cell biology, from basics to applications such as cell junction formation [34], collective cell migration [35], and even tubulogenesis [36]. When MDCK cells were isolated and cultured from canine kidney tubules, in which there was fluid flow at an osmotic flow level, the cells did not expand due to indiscriminate proliferation [37]. Most studies have rarely questioned the difference between in vivo and in vitro characteristics of MDCK cells. In this study, we found that applying a slow flow suppressed MDCK cell island expansion and peripheral cell migration. When the flow rate was set to 0.1 μL/min, cells became stabilized by maintaining cell size and density homogeneously. Still, when the flow rate was increased above 0.1 μL/min, the cells showed increased migration activity. It can be postulated that cells can sense the flow rate and react by undergoing division, migration, and death to regulate the homeostasis of a group of cells [38,39].

The analysis of cell–cell and cell–substrate physical interactions of cells within clusters provides quantitative information to understand the dynamic behavior of cell collectives. Here, we observed changes in traction and tension of cell islands exposed to the flow rate of the interstitial fluid level over time and space, compared to the static condition. It was seen that interstitial flow decreased cell traction, increased tension, and evenly distributed the cellular forces spatiotemporally more than when there was no flow. This suggests that interstitial fluid may induce homeostasis to maintain monolayer integrity by increasing the cell–cell tension instead of reducing the cell–substrate traction between cell migration and expansion.

Many studies on the effect of shear flow have reported that cells detect and respond to fluid flow by bending cilia [40] and opening ion channels [41] on the cell surface. In both pathways, the fluid flow mechanically introduces calcium ions into the cells. Thus, in this study, the calcium influx inhibitor, RR, was applied in an environment with interstitial flow to confirm the role of calcium influx. As a result, inhibition of calcium influx resulted in increased cell island area and movement speed as well as fluctuating traction and tension under interstitial flow. Interestingly, cells treated with calcium blocker under slow flow showed a similar level of traction and lower level of tension compared with the static condition. It is clear that the inhibition of calcium influx affects cell migration, leading to higher migration activity, although the basic mechanism of increased migration needs to be further investigated.

We believe that these findings suggest the role of interstitial flow as a key modulator of homeostasis in the body.

## Data Availability

Data is contained within the article and supplementary material.

## Supplementary Materials

The following are available online at https://www.mdpi.com/1996-1944/14/4/935/s1, Figure S1: Evaluation of fluid flow within the microfluidic device by multi-physics simulation, Figure S2: Average and mid-quartile range of the tension of cell islands at 0.1 and 1 μL/min flow rate for 10 h, and Table S1: Parameters used for the laminar flow simulation.
