# Supplementary material for: Study on the Expansion Dynamics of MDCK Epithelium by Interstitial Flow Using a Traction Force-Measurable Microfluidic Chip
Source: Materials (Basel). 2021 Feb 16;14(4):935. doi: 10.3390/ma14040935 (PMC7920282; doi:10.3390/ma14040935)
Supplement: Supplementary file 1 [file materials-14-00935-s001.pdf]

materials MDPI

Supplementary Materials

Study on the Expansion Dynamics of MDCK Epithelium by Interstitial Flow Using a Traction Force-Measurable Microfluidic Chip

Mirim Kim †, Hwanseok Jang †,‡ and Yongdoo Park *

Department of Biomedical Sciences, College of Medicine, Korea University, Seoul 02841, Korea; mrkmoya@korea.ac.kr (M.K.); kevin14@korea.ac.kr (H.J.)
* Correspondence: Correspondence: ydpark67@ korea.ac.kr; +82-2-2286-1460
† Authors contributed equally to this work.
‡ Present address: Laboratory of Stem Cell Bioengineering, Institute of Bioengineering, School of Life Sciences and School of Engineering, École Polytechnique Fédérale de Lausanne (EPFL), Lausanne, 1015 Vaud, Switzerland

1. Evaluation of Fluid Flow within the Microfluidic Device by Multi-Physics Simulation

In order to evaluate whether a uniform flow rate can be applied to the location of the cell islands according to the design of the chip, the flow rate was confirmed using the Navier-Stokes equation-based laminar flow module of a multi-physics simulation. The parameters for the simulation were used as shown in Table S1. The simulation was performed using the geometry and length of the microfluidic device, as shown in Figure S1(a). The fluid velocity was higher at the center than at both edges of the microfluidic chip (Figure S1(b and d)). The area within -1.75 ~ 1.75 mm (black circles) where the cell islands are located showed a difference in flow velocity of less than 5%, indicating that a uniform flow velocity over each cell island was generated. As a result of calculating the wall shear stress (tau) in the microfluidic device at a flow rate of 1 μL/min, it was confirmed that the shear stress in the cell located regions was less than 6E-5 dyn/cm^2 (Figure S1(c and e)). This result indicates that the microfluidic chip used in this study can uniformly apply a weak fluid at the level of interstitial flow to each cell island.

Table 1. Parameters used for the laminar flow simulation.

Parameter	Value	Unit	Reference
DMEM fluid density	990	kg/m3	[1]
DMEM fluid dynamic viscosity	0.00078	Pa·s	[2]
Inlet pressure	0	Pa	
Outlet flow rate	1	μL/min	

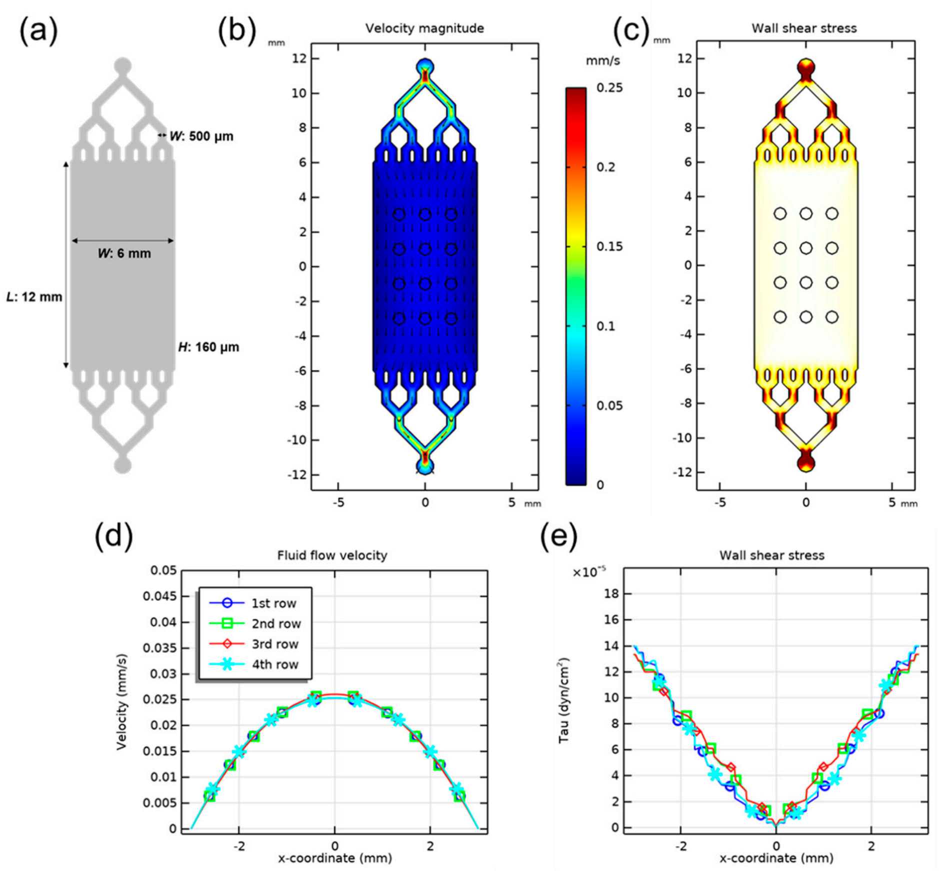

Figure S1. The velocity of the fluid flow and wall shear stress in the microfluidic device. (**a**) Dimensions of microfluidic channel (length: 12 mm, width: 6 mm, height: 160 μm) (**b**) Velocity field with vector (black arrow). Black circles indicate the locations of the cell islands. (**c**) The velocity distributions of each row location of the cell island array. (**d**) Wall shear stress field. Black circles indicate the locations of the cell islands. (**e**) The wall shear stress distributions of each row location of the cell island array.

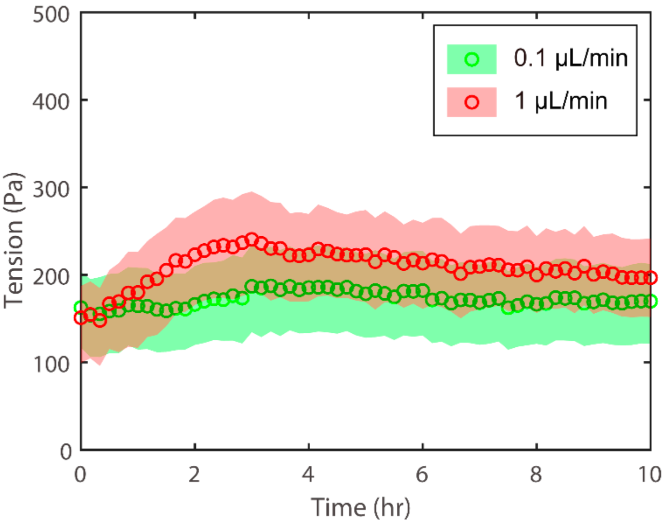

Figure S2. Average (circle) and mid-quartile range (colored field) of the tension of cell islands at 0.1 and 1 μL/min flow rate for 10 hours. (n=3).

References

1. Cimetta, E.; Cannizzaro, C.; James, R.; Biechele, T.; Moon, R.T.; Elvassore, N.; Vunjak-Novakovic, G. Microfluidic device

generating stable concentration gradients for long term cell culture: application to Wnt3a regulation of beta-catenin signaling. *Lab Chip* **2010**, 10, 3277-3283, doi:10.1039/c0lc00033g.

2. Bacabac, R.G.; Smit, T.H.; Cowin, S.C.; Van Loon, J.J.; Nieuwstadt, F.T.; Heethaar, R.; Klein-Nulend, J. Dynamic shear stress in parallel-plate flow chambers. *J Biomech* **2005**, 38, 159-167, doi:10.1016/j.jbiomech.2004.03.020.
